# Relationship between the mRNA Expression Levels of Calpains 1/2 and Proteins Involved in Cytoskeleton Remodeling

**DOI:** 10.32607/actanaturae.10947

**Published:** 2020

**Authors:** G. V. Kakurina, E. S. Kolegova, E. E. Shashova, O. V. Cheremisina, E. L. Choynzonov, I. V. Kondakova

**Affiliations:** Tomsk National Research Medical Center of the Russian Academy of Sciences, Tomsk, 634009 Russia

**Keywords:** cytoskeleton remodeling, cell motility, gene expression of actin-binding proteins, squamous cell carcinoma of the larynx and laryngopharynx, metastasis

## Abstract

Remodeling of the cytoskeleton underlies various cellular processes, including those associated with metastasis. The role of the proteases and proteins involved in cytoskeletal reorganization is being actively studied. However, there are no published data on the relationship between the mRNA expression levels of calpains 1/2 (CAPN 1/2) and the proteins associated with cytoskeleton remodeling. Therefore, the purpose of our study was to establish the relationship between the mRNA expression levels of CAPN 1/2 and the proteins involved in cytoskeletal reorganization, such as cell motility markers (SNAI1, VIM, and RND3) and actin-binding proteins (CFN1, PFN1, EZR, FSCN1, and CAP1) using the model of laryngeal/laryngopharyngeal squamous cell carcinoma (LC). The gene expression level was determined by reverse transcriptase real-time PCR and calculated using the 2-ΔΔCt method in paired tissue samples of 44 patients with LC (T1-4N0-2M0). The patients were divided into two groups: those with low and those with high CAPN 1/2 expression levels. It was found that metastasis in LC patients was associated with decreased expression levels of VIM and CAP1, and increased levels of CAPN1. A high level of CAPN2 was accompanied by a high expression level of EZR, indicating the activation of invasion processes. The results obtained need to be confirmed in further studies using a larger sample of patients and target genes. Our study is important in elucidating the mechanisms that underlie cancer progression and metastasis, a development that could subsequently open the way to a search for new prognostic and predictive markers of laryngeal/laryngopharyngeal cancer progression.

## INTRODUCTION

The high proliferation and migration potential of tumor cells is closely related to changes in the membrane mechanics associated with cytoskeletal remodeling. Detailed study of the processes of cytoskeletal rearrangement is very important in understanding the mechanisms of tumor growth. Various proteins participate in the processes of cytoskeletal reorganization. These are regulatory proteins (transcription factors: Snail, Slug, ZEB, and Twist); signaling proteins (small GTPase RhoA family, protein kinase B family, etc.); adhesive molecules; proteases (metalloproteases, calpains, proteasomes, etc.); and cytoskeleton-associated proteins (intermediate filament proteins (vimentin, keratins, etc.), actin-binding proteins (ASB), etc.) [1, 2, 3, 4]. Calpains, intracellular Ca^2+^-dependent cysteine proteases, are known to be involved in the regulation of cytoskeletal remodeling. Calpains have a wide range of substrates: cytoskeletal proteins, transcription factors, and various enzymes; their directed inhibition can be considered similar to blocking of various signaling pathways [3, 4]. Actin dynamics are regulated by a large family of actin-binding proteins (ABPs), which act as calpain substrates. The contribution of the ABPs engaged in actin cytoskeleton remodeling to tumor progression and metastasis is of fundamental importance [5-8]. Recently, the role of the tandem of ABPs and proteases in the process of tumor growth has been extensively investigated [7, 8]. We aimed to study the relationship between the expression levels of CAPN1/2 and the genes of the cell motility protein (SNAI1, VIM, RND3) and actin-binding proteins of cofilin (CFN1), profilin (PFN1), ezrin (EZR), fascin (FSCN1), and adenylyl cyclase-associated protein 1 (CAP1) using a model of highly aggressive laryngeal and laryngopharyngeal cancer (LC).

## MATERIALS AND METHODS

Paired samples of tumor and morphologically unchanged laryngeal and laryngopharyngeal tissues obtained during videolaryngoscopy from 44 patients admitted for treatment at the Oncology Research Institute were used. Laryngeal squamous cell carcinoma was histologically verified (T1-4N0M0 – 21, T1-4N1M0 – 16, T1-4N2M0 – 7). The median age of the patients was 56 ± 7 years. The study was carried out in compliance with the principles of voluntariness and confidentiality in compliance with the Fundamentals of the Legislation of the Russian Federation on Protection of Public Health; permission was secured from the Institute’s Ethics Committee. Tissue samples were stored in a RNAlater solution (Ambion, USA) at –0°C.

**Methods**

Total mRNA was isolated using the CCR-50 kit (Biosilica, Novosibirsk, Russia) in accordance with the manufacturer’s protocol. The quality of the mRNA was evaluated using capillary electrophoresis and TapeStation instruments (Agilent Technologies, USA) and an R6K ScreenTape kit (Agilent Technologies, USA). cDNA was obtained on the mRNA matrix using a set of reagents for reverse transcription OT-1 (Syntol, Moscow, Russia) according to the manufacturer’s protocol. The level of mRNA expression was evaluated by real-time PCR (RT-qPCR) using the SYBR Green detection method on 96-well plates with the Bio-Red iCycler thermocycler (Bio-Rad, USA) and was calculated using the 2-ΔΔCt method. Primers were selected using the Vector NTI Advance 11.5 software and the NCBI database. The primer sequences are given in *Table* [7]. The “household” GAPDH gene was used as a reference gene. The reaction products were evaluated using capillary electrophoresis and TapeStation instruments (Agilent Technologies, USA); the melting curve analysis (Rotor Gene 6000) was also used.

**Statistical analysis**

Statistical analysis was conducted using nonparametric criteria: the Mann–Whitney U and Kruskal–Wallis tests, as well as Spearman’s and Kendell’s rank correlation coefficients. The results are shown as M ± m, where M is the mean, m is the standard deviation, and n is the number of patients in the group.

## RESULTS AND DISCUSSION

The patients were divided into groups as follows: with a low expression level of CAPN1 (L-CAPN1): from 0.0 to 5.1 (n = 24) and a high expression level of CAPN1 (H-CAPN1): from 5.03 and higher (n = 20); and with a low expression level of CAPN2 (L-CAPN2): from 0.0 to 2.0 (n = 23) and a high expression level of CAPN2 (H-CAPN2): from 2.01 and higher (n = 21). The high expression level of CAPN1 in the sample of LC patients was associated with the presence of regional metastases (r = 0.4, *p *≤ 0.05), and the relationship between the expression level of CAPN2 and the metastatic status was insignificant (r ≥ 0.3, *p *= 0.055). We can assume that an increase in the sample size of LC patients would reveal a significant association of CAPN2 expression with metastasis.

An analysis of the expression of the SNAI1, RND3, and VIM genes encoding cell motility markers demonstrated that LC patients with increased expression of CAPN1 had a significantly reduced VIM expression level (*Table 1*). In LC patients with metastases, the mRNA expression level of SNAI1, RND3, and VIM tended to increase. The results obtained were not consistent with prior studies [6]. This may be explained by the features of metastasis in the LC patients. Therefore, further studies are needed.

**Table 1:** Association between the mRNA expression levels of the genes encoding cell motility markers and the mRNA expression levels of the genes encoding proteases

Gene	L–capn1	H–capn1	P1	L–capn2	H–capn2	P2
SNAI1	3.89 ± 5.09	0.43 ± 0.54	0.11	2.56 ± 5.28	3.54 ± 7.17	0.88
VIM	25.31 ± 12.6	6.8 ± 11.08	0.04	19.26 ±2 5.89	22.52 ± 34.62	0.98
RND3	5.7 ± 11.8	20.33 ± 26.59	0.28	19.88 ± 43.44	11.72 ± 21.60	0.54

Note:

The P1 value is a statistically significant difference between L–CAPN1 and H–capn1.

The P2 value is a statistically significant difference between L–CAPN2 and H–capn2.

Comparison of the expression levels of the actinbinding proteins and proteases (*Table 2*) revealed that the expression level of CAP1 was significantly decreased, while the expression level of CAPN1 was increased.

**Table 2:** The mRNA expression level of the genes encoding the proteins associated with cytoskeletal remodeling with respect to the expression level of proteases

Gene	L–capn1	H–capn1	P1	L–capn2	H–capn2	P2
FCSN1	5.06 ± 8.19	12.91 ±2 4.56	0.52	11.45 ± 21.34	5.96 ± 9.01	0.93
EZR	16.37 ± 24.03	17.31 ± 45.88	0.56	8.92 ± 17.61	31.38 ± 13.96	0.02
PFN1	6.95 ± 12.23	11.76 ± 22.59	0.90	24.30 ± 45.43	7.12 ± 9.44	0.74
CFN1	19.57 ± 25,75	16.67 ± 28.61	0.39	14.28 ± 25.32	21.64 ± 26.97	0.26
CAP1	20.30 ± 29.7	9.97 ± 16.2	0.05	18.70 ± 32.08	14.16 ± 24.26	0.54

Note:

The P1 value is a statistically significant difference between L–CAPN1 and H–capn1.

The P2 value is a statistically significant difference between L–CAPN2 and H–capn2.

The decrease in the expression level of CAP1 in LC samples was not consistent with previously obtained results according to which the CAP1 level increases with tumor growth [7]. The role played by CAP1 in tumor growth has not been established yet [7]. There is also no evidence of coexpression of this protein and proteases. In the group of patients with a high expression level of CAPN2, high expression of the gene encoding esrin, the calpain substrate, was established [8]. However, in our group of patients with a high CAPN2 expression, a 3.8-fold increase in the EZR expression (unrelated to CAPN1 expression) was observed. The high expression levels of esrin and CAPN2 were associated with a high assembly/disassembly rate of focal contacts [8]. Moreover, ezrin was shown to be required for the calpain-mediated proteolysis of talin, focal adhesion kinase, and cortactin [8]. The coexpression of CAPN2 and EZR established by us can be an indication that the rate of association/dissociation processes in the focal contacts is increased, attesting to the acquired mobility of tumor cells. It should be noted that the high level of ezrin is associated with a low survival rate of cancer patients [9]. Today it seems impossible to assess whether a relationship exists between CAPN2 and EZR coexpression and LC metastasis. This mechanism is likely to be involved in other processes; in particular, in the case of an invasion of tumor cells.

In our study we established moderate correlations between the VIM and CAP1, EZR and CAPN2, and PFN1 and CFN1 expressions (r ≥ 0.6, *p *≤ 0.05) in LC patients with reduced CAPN1 expression.

In the cases with increased CAPN1 expression, correlations between the SNAI1 and PFN1, VIM and RND3, and CAP1 and CFN1 expressions were established (r ≥ 0.6, *p *≤ 0.05). Given that a high level of CAPN1 expression is mainly associated with the presence of regional metastases (r = 0.4, *p *≤ 0.05), we can assume activation of the epithelial–mesenchymal transition. Changes in the CAPN2 expression altered the relationships between the expressions of the genes involved in cell motility. In the cases with a low level of CAPN2 expression, moderate correlations between the expression levels of FSCN1 and PFN1, as well as between PFN1 and CFN1, were observed (r ≥ 0.6,* p *≤ 0.05). In the cases with a high level of CAPN2 expression, moderate correlations between the expression levels of CAP1, PFN1 and CFN1 were established (r ≥ 0.6, *p *≤ 0.05). Thus, the level of CAPN2 expression in our sample of LC patients correlated with the expression profile of ABPs and was not associated with the metastatic status (r ≥ 0.3, *p *= 0.055). The expression levels and significant correlations of calpains 1 and 2 with clinical and morphological characteristics likely depend on other characteristics of the tumor [10], or are associated with the important role played by other members of the calpain family in the pathogenesis of LC [11, 12].

A tumor’s metastatic potential is determined by the ability of tumor cells to acquire atypical morphofunctional and molecular genetic properties, leading to uncontrollable growth and proliferation, as well as to the emergence of a locomotor phenotype.

The mobility of tumor cells is associated with a transformation of the actin cytoskeleton, which is accompanied by changes in the composition, functions, and activity of various proteins and their genes [2, 6–8]. Our results indicate that laryngeal cancer metastasis is associated with high CAPN1 and low VIM and CAP1 expression levels. The decrease in VIM expression is somewhat contrary to the accepted norms and requires a more careful verification with the inclusion of a larger cohort of patients and target genes that can be involved in alternative ways of regulating metastasis. The expression level of CAPN2 was not significantly associated with lymphogenous metastasis in our sample of LC patients. A high level of this protease was accompanied by a high level of EZR expression, which may be indirect attestation to the activation of invasion processes [9, 10]. In general, our results were confirmed by data on the tissue specificity of calpains (e.g., on the relationship between laryngeal cancer and CAPN10 [11]); the expression level of CAPN6 negatively correlated with the disease outcome in patients with head and neck squamous cell carcinoma [12]. Nevertheless, the results of our study demonstrate that detailed research into the molecular mechanisms of the interplay between the calpain system and the proteins associated with cytoskeletal remodeling at the transcriptome and proteomic levels is needed.

## CONCLUSION

In our sample of LC patients, we established a relationship between the expression levels of CAPN1 and cytoskeletal proteins such as vimentin and CAP1, which are involved in cytoskeleton remodeling. The expression level of CAPN2 correlated with the expression of ezrin mRNA, a linker protein between the plasma membrane and actin cytoskeleton, whose high level was associated with invasive growth [10]. Lymphogenous metastasis in LC patients correlated with a high level of CAPN1 expression, while the CAPN2 level did not show such a relationship. The results obtained indicate that further studies need to be conducted using larger samples of patients and an expanded panel of target genes that can theoretically participate in the tandem of calpain–cytoskeletal proteins. The data obtained are important for elucidating the mechanisms underlying cancer progression and metastasis, which subsequently can open the way for a search for new prognostic and predictive markers of laryngeal/laryngopharyngeal cancer progression.

## Abbreviations

CAP1: adenylyl cyclase-associated protein 1
L/L SCC: squamous cell cancer of larynx and laryngopharynx
ABP: actin-binding proteins
CFN1: cofilin
PFN1: profilin
EZR: ezrin
FSCN1: fascin
CAPN 1: calpain 1
CAPN 2: calpain 2
